# Micro and Nano-plastic particles: What are they and do they effect cardiovascular health?

**DOI:** 10.3126/nje.v14i1.64183

**Published:** 2024-06-30

**Authors:** Indrajit Banerjee, Jared Robinson, Brijesh Sathian, Indraneel Banerjee, Edwin R van Teijlingen, Bedanta Roy, Ashok Pratap Singh

**Affiliations:** 1 Sir Seewoosagur Ramgoolam Medical College, Belle Rive, Mauritius; 2 Geriatric and long term care Department, Rumailah Hospital, Hamad Medical Corporation, Doha, Qatar; 3 Penn Highlands Healthcare, Pennsylvania, United States of America; 4 Centre for Midwifery& Women’s Health, Bournemouth University, Bournemouth, UK; 5 Quest international University, Perak, Malaysia

## Background

Microplastics and nanoplastic particles (MNPs) have become ubiquitous in the environment. As the world entered the twenty-first century and technology advanced, the use of plastics, primarily single-use plastics (which are polymeric materials), rapidly increased to meet the global demand based on our lifestyles and expectations of comfort [1]. The use of these polymeric compounds has been instrumental in building a world in which we currently find ourselves. The innate characteristics of plastics have ensured that these polymeric compounds are used on a global basis and have penetrated almost every industry and market, ranging from agriculture to construction and pharmaceuticals [2]. As time has passed and technology has progressed, our understanding of these polymeric compounds, how they interact with our environment, and how they affect our health has improved. A substantial dearth of knowledge surrounding these substances and the natural environment still exists [3]. As with all technological innovations there are a range of unintended, and often negative, consequences of MNPs, including the fact that MNPs are now found in the deepest psrts of our oceans and on the highest mountains in the Himalayas. The negative polluting and toxic effects of these microplastics are becoming more apparent, and there are many ways in which these MNPs affect our broader living environment as well as our bodies. We are beginning to understand the the physical and mechanical properties of microplastics via thrombus formation and immune upregulation, coupled with interference with translocation and inflammation, all of which are proposed as physical mechanisms. It is also understood that toxic effects of NMPs are linked to the leaching of chemicals and toxins into native cellular tissue, which causes damage to the organs involved [4].

### What are MNP’s

For a piece of plastic to be considered a “microplastic,” it must be less than 5 mm in length, and a nanoparticle is defined as a piece that ranges from 1 to 1000 nm in size [5]. Microplastics” have two origins and can be classified accordingly [6]. Primary microplastics are originally less than 5 mm in size for use in the compounding of cosmetics and other polymeric compounds, whereas secondary microplastics are originally larger, but a byproduct of larger bodies of plastic that have been broken, eroded, or shaved off. The innate issue with these microscopic pieces of plastic is that they pollute and contaminate every inch of our planet, from our oceans to the food we eat, and even our own bloodstream. The various forms of exposure to MNPs range from dermal ingestion to inhalation [7]. A study undertaken by the Vrije University of Amsterdam (the Netherlands) found that nearly 80 percent of the samples of meat and dairy products from farm animals tested contained MNPs. Therefore, it is widely evident that the majority of packaged meat and dairy products consumed on a daily basis are contaminated with MNPs. The ubiquitous nature of MNPs in our environment poses a threat, both directly and indirectly, to the human race. The majority of these plastic polymeric compounds are produced from petrochemicals and contain innate substances and toxins that contaminate organic living matter [8]. A further drawback of these polymeric compounds is the fact that they are extremely hardy, take anywhere between 100 and 1000 years to decompose, and, in doing so, produce countless MNPs during this decomposition process. The plastics we produce on our planet will harm not only us but also future generations for thousands of years [9].

### Current human studies

Several studies have provided evidence that MNPs have a negative effect on the cardiovascular system, primarily through microvascular toxicity [10]. New findings not only show the mere presence of these MNPs in atheromas and within the cardiovascular system but also provide evidence that the presence of these MNPs results in poorer outcomes for patients. For example, a multicentric study conducted by Marfella et al. on 304 asymptomatic end-atherectomy patients analyzed resected plaque specimens via pyrolysis-gas chromatography-mass spectrometry, electron microscopy, and stable isotope analysis. The two main microplastic compounds identified in the 150 patients were polyvinyl chloride (one of the most common plastic substances found on earth) and polyethylene. Electron microscopic findings further supported the chemical analysis, with the presence of jagged foreign body particles (plastics) surrounded by inflammatory macrophages being noted in these atheroma’s. This alludes to the inflammatory basis and drive by which these plastics exacerbate and thereby worsen the formation and development of atheromas. Furthermore, the study concluded that patients suffering from the presence of microplastics within their atheromas had a higher likelihood of suffering from a primary endpoint event such as cerebrovascular apoplexy or myocardial infarction [11,12].

### Animal model evidence

Human studies provide evidence of the toxic effects of MNPs. Evidence from animal models also strongly supports the notion of toxicity evoked by MNPs. A study performed by Zhang et al. on mice after chronic exposure to microplastics showed damage to the cardiac tissue, with increased fibrosis and lipid accumulation in the myocardial tissue. It was also noted that the proposed underlying mechanism and mediator behind this cardiac toxicity were transcriptome-wide m6A modifications. Further studies on the exact mechanism underlying cardiac toxicity are required [13,14].

Proposed mechanisms of MNP’s on the cardiovascular system The mechanism underlying the effect of these MNP’s on critical cardiovascular events is poorly understood, but it is believed that their effect ranges from interference with haematological coagulation profiles, thrombus formation, platelet aggregation, immune regulation, inflammation, microvascular toxicity, and micro-thrombosis. The full extent of the pathological and physiological disruptions caused by these MNPs is yet to be discovered and poorly understood. Animal studies have shown that m6A modification across the transcriptome is the main cause and mediator of heart damage [15].

### Mitigating MNPs in our natural environment

As we are becoming more aware of the long-term effects of these MNPs and the effect of these plastics on both our health and the health of the environment around us, it is becoming more pertinent to reduce and mitigate the production and subsequent spread of these MNPs [16]. Many campaigns and eco-friendly nonprofit organizations have begun this trend [17]. New legislation and stricter enforcement thereof will be instrumental in reducing the production and release of MNPs [18]. These microplastics are classified as food adulterants and thereby allow judicial support to further tighten the production thereof. Each individual plays a role by reducing the use of single-use plastics, opting for reusable organic and natural products that are biodegradable and eco-friendly; for example, the use of new “bioplastics” developed from coconut husks is a logical and safer choice when compared to traditional polymeric compounds. Furthermore, clean-up programs and newer technologies can be developed to aid the removal of MNPs from oceans and the atmosphere [17].

## Conclusion

It is evident that MNPs have a detrimental effect on cardiovascular health, the full extent of which is unclear. Further large-scale studies are required to provide improved insight into legislation and governing action. It is suspected that the extent to which MNPs affect human physiology and cardiovascular health will be further extrapolated with such studies, and the degree to which these MNPs affect human physiology will be worse than what is currently understood and anticipated. Until these studies have been conducted, it is advised that the use of plastics in all aspects of our daily lives should be minimized, with greater emphasis placed on the use of biodegradable and eco-friendly products.
